# Morphological variation in the anterior cranial fossa

**DOI:** 10.1002/cre2.163

**Published:** 2019-01-31

**Authors:** Emi Kasai, Shintaro Kondo, Kazutaka Kasai

**Affiliations:** ^1^ Department of Orthodontics and Dentofacial Orthodontics, School of Dentistry at Matsudo Nihon University Chiba Japan; ^2^ Department of Anatomy, School of Dentistry at Matsudo Nihon University Chiba Japan

**Keywords:** computed tomography, cranial base, ethmoid bone, frontal bone, sphenoid bone

## Abstract

The anterior cranial fossa is an important anatomical landmark in clinical orthodontics consisting of the frontal, ethmoid, and sphenoid bones. The relationships between these bones remain poorly understood. The purposes of the present study were to describe the morphological relationships among the three bones and to discuss the factors contributing to individual variations in adult skulls based on postnatal development. Skulls of 100 Indian adults and 18 Japanese juveniles were observed both macroscopically and using computed tomography images in the median sagittal plane. Three types of relationship were seen among the three bones in adult skulls: (a) a triangular border between ethmoid and sphenoid bones (ethmoid spine), (b) a straight or concave border between ethmoid and sphenoid bones, and (c) frontal bone lying between the ethmoid and sphenoid bones. In the juvenile skull, structures corresponding to those in adults were observed. These three bones comprise the anterior cranial base, each with differing developmental processes, and slight differences in these processes seem to be reflected in the morphological variations seen among adults.

## 1 INTRODUCTION

The cranial base is used as a standard plane for cephalometric analysis in clinical orthodontics (Downs, 1948; Proffit, Fields, & Sarver, 2007; Steiner, 1953). It is important to elucidate individual variation of the measurement landmarks in the cranial base. The cranial base is divided into anterior, middle, and posterior cranial fossae, which exhibit forms corresponding to the structure of the brain (Standing, 2015). Although the cranial base mainly develops by endochondral ossification, the details vary depending on the site. As for the bones consisting anterior cranial fossa, the ethmoid and sphenoid bones are formed by endochondral ossification, but the frontal bone is formed by intramembranous ossification extending from the calvaria (Sperber, 2010).

The anterior cranial fossa is formed by the orbital part of the frontal bone, the cribriform plate and crista galli of the ethmoid bone, and the lesser wings and anterior part of the body (jugum sphenoidale and prechiasmatic sulcus) of the sphenoid bone (Standing, 2015). Three of these bones are connected by sutures (the spheno‐frontal, spheno‐ethmoidal, and fronto‐ethmoidal sutures) and in some cases by a synchondrosis (spheno‐ethmoidal synchondrosis). The anterior cranial fossa is thought to grow by both intersuture and cartilaginous growth.

**Figure 1:**
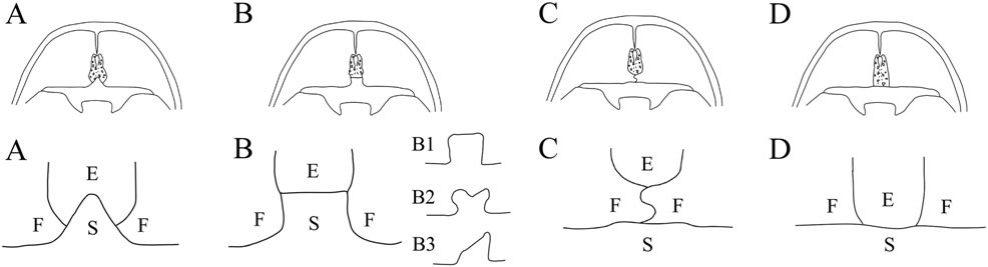
Structures of the anterior cranial fossa cited in anatomical textbooks. (A) Triangular protrusion of the sphenoid body (ethmoidal spine) projecting towards the ethmoid bone (Gray, 1973; Hansen, 2010; Morris, 1966; Rohen, 1973; Sobotta, 2006). (B) Anterior border of the median part of the protrusion of the sphenoid body is short and straight, with suture between the ethmoid and sphenoid bones (Snell, 1978); van der Linden and Enlow (1971) subdivided B into three: B1, symmetrical form with straight anterior medial border; B2, symmetrical form with concave anterior outline; and B3, asymmetrical form. (C) Frontal bone intervenes between the ethmoid and sphenoid bones (Grant, 1972; Moore, 2004). (D) Anterior border of the sphenoid body is straight (van der Linden & Enlow, 1971). E: ethmoid bone; F: frontal bone; S: sphenoid bone

In the typical textbooks of anatomy (Drake, Vogl, Mitchell, Tibbitts, & Richardson, 2015; Grant, 1972; Gray, 1973; Hansen & Netter, 2010; Moore & Dalley, 2004; Morris, 1966; Rohen, 1973; Snell, 1978; Sobotta, 2006), these three bones are described as showing three patterns (Figure 1, A–C), but the descriptions vary between authors. To further complicate the matter, van der Linden and Enlow (1971) reported significant individual variation in the sutures of the anterior cranial fossa (Figure 1, B1–B3) and also added another pattern (Figure 1, D). These descriptions showed differences from those in previous anatomical textbooks and suggest that further investigation of the individual variation in the anterior cranial fossa is required or whether the descriptions can be standardized because morphological characteristics have not been sufficiently analyzed. Because the floor of the anterior cranial fossa comprises three bones, the possibility exists that morphological variations in adult skulls may arise from individual differences in the growth patterns of these bones.

The aim of this study was to investigate individual variations in the anterior cranial fossa in adult skulls and, in addition, to consider variation during growth by observing skulls from childhood, to investigate factors influencing individual variations in adult skulls.

## 2 MATERIALS AND METHODS

### 2.1 Materials

#### 2.1.1 Adult skulls

We observed 100 adult skulls from Indian individuals stored in the Department of Anatomy, School of Dentistry at Matsudo, Nihon University. Exact ages and sexes could not be identified because whole‐body skeletons and personal information were lacking, so sexes were combined for analysis. In all individuals, the second molar teeth had completely erupted, the third molar tooth often erupted, and extent attrition of the permanent teeth was also advanced. There were no individuals remaining spheno‐occipital synchondrosis. From these information, we inferred that these individuals were fully adults. The ethmoid, sphenoid, and frontal bones remained unbroken and could be observed in all of these skulls.

#### 2.1.2 Skulls in developmental stage

We observed 18 juvenile skulls from Japanese individuals, ranging in age from 4 months to 17 years old, stored in the University Museum at the University of Tokyo (Table 1). Because there are few specimens that could be observed, we showed results with both sexes combined.

**Table 1:** Numbers of specimens by developmental stage

Age	Male	Female	Unknown
4–6 months	1	4	
2 years			1
7 years	1	1	
10–17 years	5	5	
Total	7	10	1

### 2.2 Methods

Skulls were analyzed by macroscopic observation with the naked eyes and computed tomography (CT) images in the median sagittal plane. Each of the 100 adult skulls was analyzed by macroscopic observation, and 44 skulls were also observed by CT. All skulls in the developmental stages were also analyzed by macroscopic observation and micro‐CT images.

The relationships and shapes of the ethmoid, sphenoid, and frontal bones were observed macroscopically with reference to the literature. We observed the relationship between sphenoid and ethmoid bones, in terms of whether these bones were in contact with each other. When contact was present, the status of the relationship between the two bones was either fused or sutured. When the bones were not in contact, the frontal bone had intervened between the ethmoid and sphenoid bones. We paid special attention to observing the shape of the ethmoidal spine on the sphenoid bone. On CT images, we confirmed whether the three bones were in contact with each other in terms of internal configuration.

Imaging conditions for CT were as follows. Adult skulls were scanned using dental cone‐beam CT (KaVo 3D eXam+; KaVo Dental Systems Japan, Tokyo, Japan) at the School of Dentistry at Matsudo, Nihon University. Each cranium was scanned once, under the following scan parameters: tube voltage, 120 kV; current, 5 mA; and voxel, 0.3 mm. Skulls in developmental stages were scanned using a micro‐CT scanning system (TXS225‐ACTIS; TESCO, Tokyo, Japan) housed at the University Museum of the University of Tokyo. Each cranium was scanned once, under the following scan parameters: tube voltage, 130 kV; current, 0.30 mA; and slice thickness and increment, 0.35–0.45 mm. Skulls in developmental stages were imaged with micro‐CT system to scan higher resolution than the adults.

This study conducted research in accordance with the Declaration of Helsinki in order to target human bones but was not subject to ethical review.

## 3 RESULTS

### 3.1 Adult skulls

**Figure 2:**
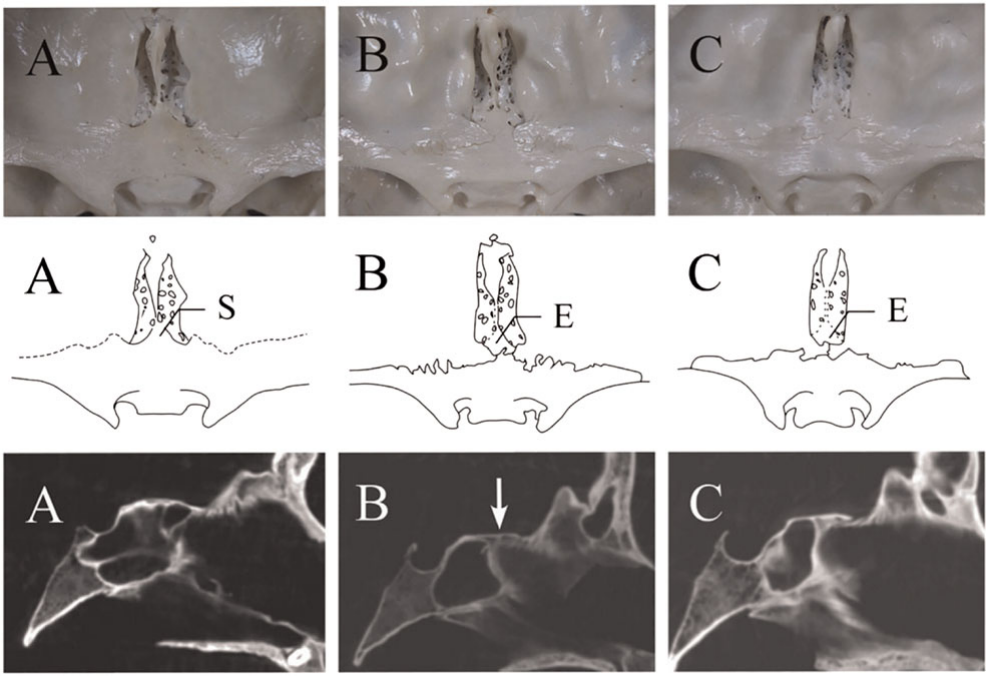
Morphological variations of the anterior cranial fossa in adult skulls. Upper row shows endocranial photographs, middle row shows traced images, and lower row shows dental cone‐beam computed tomography (CT) images in the median sagittal plane. (A) Triangular protrusion of the sphenoidal body projecting towards the ethmoid bone. Distances from the posterior border of the ethmoid bone to the lesser wing of sphenoid bone were varied. The spheno‐ethmoidal suture is not observed on CT images. (B) The anterior border of the median part of the protrusion of the sphenoid body is straight. The spheno‐ethmoidal suture is clearly evident on CT images (arrow). (C) Frontal bone intervenes between the ethmoid and sphenoid bones. The boundary of the three bones is unclear on CT images

Three patterns were observed to clarify relationships among the ethmoid, frontal, and sphenoid bones (Figure 2). The essential points for observation were as follows:

- Relationship of the ethmoid and sphenoid bones, categorized as in contact (types A and B) or not in contact (type C); and
- shape of the projection of the sphenoid bone (ethmoid spine), categorized as showing a triangular outline (type A) and straight or concave outline (type B).

Type A showed a triangular protrusion of the sphenoid bone projecting towards the ethmoid bone (Figure 2, A). The ethmoid bone was continuous with the anterior border of the sphenoid body in the central part of crista galli. The ethmoid and sphenoid bones were not clearly distinguishable in the median part but were uneven in the lateral parts of right and left sides of the cribriform plate, with the anterior border of the ethmoid spine higher than the cribriform plate. Distance from the lesser wings of sphenoid bone to the anterior border of the sphenoid body was variable. The spheno‐ethmoidal suture was not observed.

**Figure 3:**
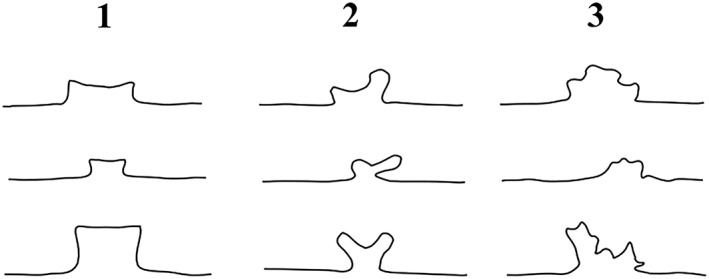
Morphological variations in spheno‐ethmoidal suture. The border is straight or slightly concave posteriorly (1), showing a concave outline (2), and nonsymmetrical or amorphous in shape (3)

Type B showed a straight or concave anterior border of the median part of protrusion of the sphenoid bone (Figure 2, B). The spheno‐ethmoidal suture was varied in shape: straight or slightly concave, concave, and nonsymmetrical, or amorphous outline (Figure 3). The posterior border of the ethmoid bone continuing to the crista galli was bulging and higher than the cribriform plate. Distance from the lesser wing to the anterior border of the sphenoid body also varied, as in type A.

Type C showed a form in which the frontal bone intervened between the ethmoid and sphenoid bones (Figure 2, C). The left and right frontal bones showed sutures at the center of the anterior cranial fossa.

Table 2 shows the sample size for each structure type of the anterior cranial fossa in adult skulls. Type A was the most frequent structure, followed by type B. Type C was rarely observed.

**Table 2:** Structure of the anterior cranial base in adult skulls

	Structure of anterior cranial base			
	A	B	C	Total
	*n* (%)	*n* (%)	*n* (%)	*n* (%)
Macroscopic observations	59 (59.0)	34 (34.0)	7 (7.0)	100 (100.0)

*Note*. Type A: Triangular protrusion of sphenoid bone projecting towards the ethmoid bone. Type B: Flattened anterior end of the median part of the protrusion of the sphenoid bone. Type C: Frontal bone intervening between the ethmoid and sphenoid bones.

Forty‐four skulls (type A, *n* = 20; type B, *n* = 17; type C, *n* = 7) were imaged by dental cone‐beam CT, and the spheno‐ethmoidal sutures were investigated in the median sagittal plane. In type A, this suture was not observed. The ethmoid bone was thus continuous with the sphenoid bone, but the ethmoid bone was overlapped by sphenoid bone (Figure 2, A). The two bones were confirmed to be fused not only superficially but also in the deep structures. In 10 cases of type B, the suture was observed, and the border between the ethmoid and sphenoid bones was clearly evident (Figure 2, B). In six cases of type B, the suture was not observed on the CT images, and the deep structure showed partial fusion. In type C, the suture was not observed, and the boundaries of the three bones were unclear because the bones displayed complicated interlocking (Figure 2, C).

### 3.2 Skulls in developmental stage

The following descriptions were made by dividing specimens into three age groups: early childhood (from 4 months to 2 years old), later childhood (7 years old), and adolescent (10–17 years old).

#### 3.2.1 Early childhood (4 months to 2 years old)

**Figure 4:**
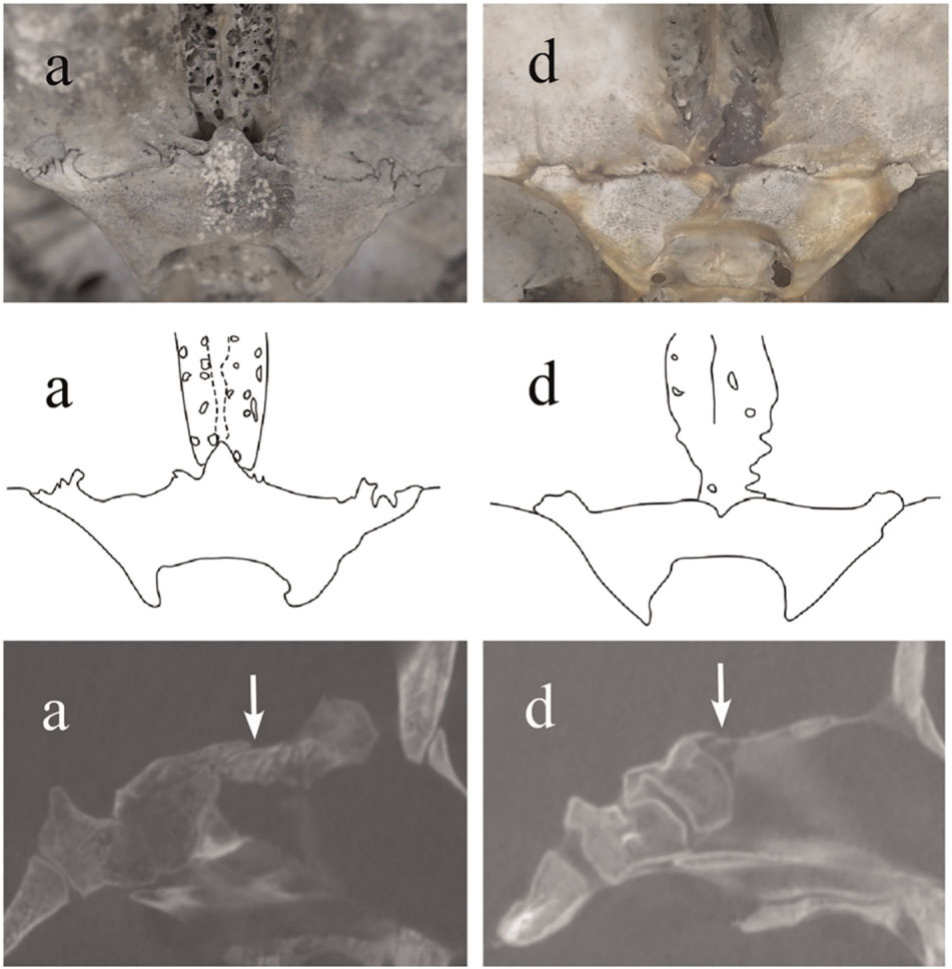
Morphological variations in the anterior cranial fossa in skulls from individuals in early childhood (4–6 months to 2 years old). Upper row shows endocranial photographs, middle row shows traced images of photographs, and lower row shows micro‐CT in the median sagittal plane. (a) Triangular protrusion of the sphenoid body; (d) anterior border of the sphenoid body is straight and/or slightly concave posteriorly. The boundary between ethmoid and sphenoid bones is clearly evident in all individuals on micro‐CT (arrows). CT: computed tomography

Two variations were seen in this stage. The first showed a triangular ethmoid spine similar to type A in adult skulls (Figure 4, a). The ethmoid bone was clearly distinguishable from the sphenoid bone. In the second variation, the anterior border of the sphenoid body showed a straight outline and/or slightly concave posterior border. The anterior border of the sphenoid body in this type did not project anteriorly, and the ethmoid and sphenoid bones were clearly distinguishable (Figure 4, d). This configuration was similar to that of type D (Figure 1, D) described by van der Linden and Enlow (1971). In adult skulls, the structures were represented by capital letters (A, B, C, and D); skulls in the developing stages were represented by small letters (a, b, c, and d).

In all individuals, the spheno‐ethmoidal suture was observed on micro‐CT. An impermeable white border was found between the ethmoid and sphenoidal bones (Figure 4, arrows). The ethmoid bone was overlapped by sphenoid bone.

#### 3.2.2 Later childhood (7 years old)

**Figure 5:**
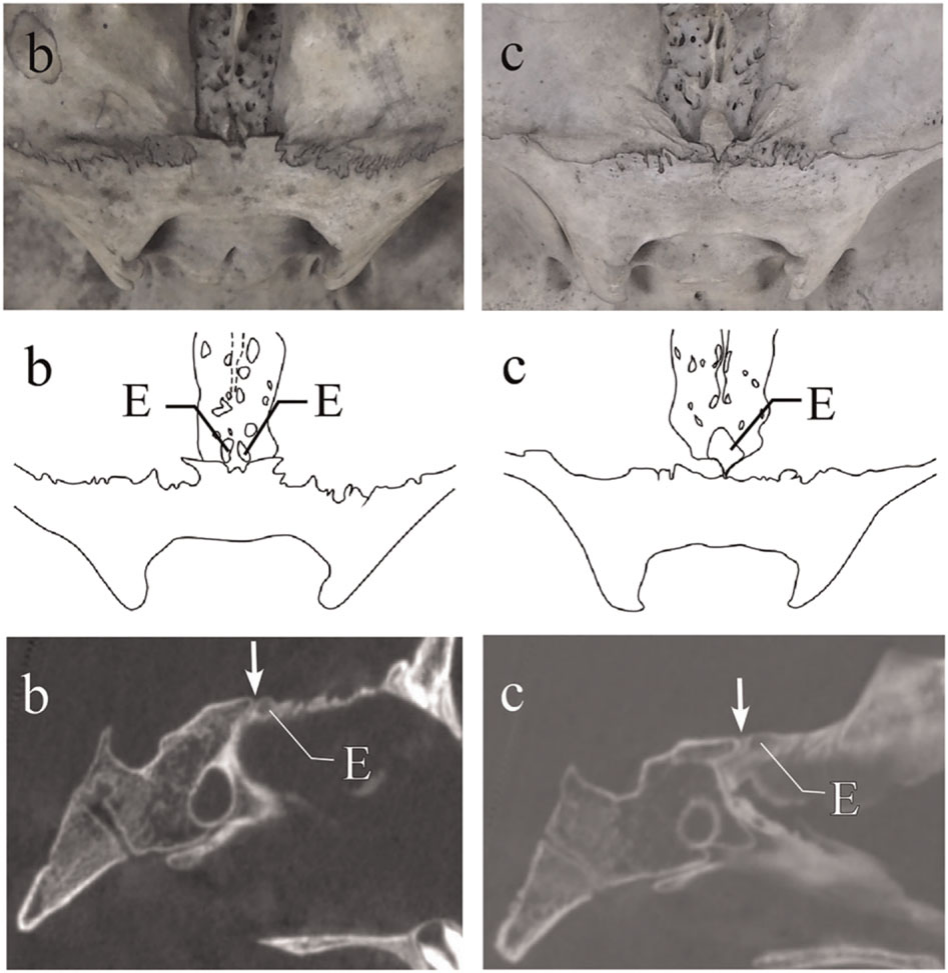
Morphological variations of the anterior cranial fossa in late childhood (7 years). Upper row shows endocranial photographs, middle row shows traced images of photographs, and lower row shows micro‐CT images in the median sagittal plane. (b) Median part of the sphenoid body projects slightly anteriorly, and anterior border of the protrusion has a straight outline. (c) Median part of the sphenoid body does not project anteriorly. Orbital plates of the right and left sides of frontal bones are close to each other in the median part. E, ethmoid bone. Arrows show spheno‐ethmoidal suture on CT images. CT: computed tomography

Samples for this stage were two individuals (Table 1). In the first skull, the median part of the sphenoid body projected slightly anteriorly, and the anterior border of the protrusion was straight (Figure 5, b). The first specimen corresponded to type B in adult skulls. In the second skull, the median part of the sphenoid body did not project anteriorly, and the orbital plate of the frontal bone on the right and left sides were close to each other in the median part (Figure 5, c). The second specimen was similar to type C in adult skulls. Orbital plates of the right and left sides of frontal bone were close to each other in the median part. They were not completely in contact, and the ethmoid bone intervened between the orbital plates. In the both skulls, the spheno‐ethmoidal suture was observed macroscopically. In the micro‐CT images, the ethmoid bone was overlapped by sphenoid bone, and the border between the two bones was also distinguishable. A small piece of bone appeared between the ethmoid and sphenoid bones. This particle looked like independent from both the ethmoid and sphenoid bones but was part of the ethmoid bone based on macroscopic and micro‐CT image observations.

#### 3.2.3 Adolescent (10–17 years old)

**Figure 6:**
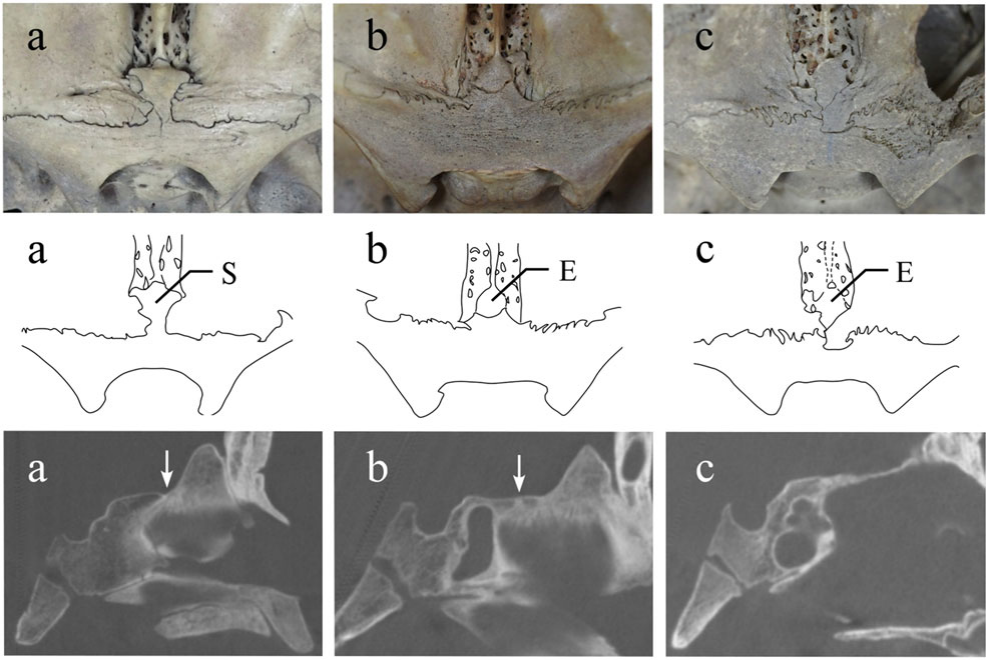
Morphological variations of the anterior cranial fossa in the skulls of adolescents (10 to 17 years). Upper row shows endocranial photographs, middle row shows traced images of photographs, and lower row shows micro‐CT in the median sagittal plane. (a) Protrusion of the sphenoid body projects anteriorly. (b) Anterior border of protrusion of the sphenoid body is slight, and posterior border of the crista galli shows budding. (c) Frontal bone is intervening between the ethmoid and sphenoid bones. Arrows show spheno‐ethmoidal suture on CT images. CT: computed tomography

Three morphological variations were observed in this stage (10 individuals). In the first, the protrusion of the sphenoid bone towards the ethmoid bone was long (Figure 6, a). In the second, the protrusion of the ethmoid spine was short and had a straight or concave anterior border (Figure 6, b). In the third variation, the frontal bone intervened between the ethmoid and sphenoid bones (Figure 6, c).

In the first two types, the spheno‐ethmoidal suture was observed on macroscopic and micro‐CT observations, and the ethmoid bone was overlapped by sphenoid bone (Figure 6, a and b). However, in the latter type, the boundary between the three bones was unclear, presumably related to the complicated interlocking on micro‐CT, and the ethmoid bone was not overlapped by sphenoid bone (Figure 6, c).

Observations for skulls in the developmental stages are summarized in Table 3. Type b was the most frequent, followed by type d and a, and type c was found two cases. Type d appeared only in early childhood.

**Table 3:** Structure of the anterior cranial base in skulls by developmental stage

Developmental stage	Structure of anterior cranial base			
	a	b	c	d
Early childhood (4–6 months and 2 years)	1			5
Late childhood (7 years)		1	1	
Adolescent (10–17 years)	3	6	1	
Total	4	7	2	5

*Note*. Type a: Triangular protrusion of the sphenoid bone projects towards the ethmoid bone. Type b: Flattened anterior end of the median part of the protrusion of the sphenoid bone. Type c: Frontal bone intervening between ethmoid and sphenoid bones. Type d: Straight anterior border between the ethmoid bone and the sphenoid body.

## 4 DISCUSSION

### 4.1 Development of the anterior cranial fossa in fetal life

The frontal bone formed by intramembranous ossification, from a pair of ossification centers forming in the region of the eyebrow arch at around 8 weeks postconception (Sperber, 2010). Three pairs of secondary calcification centers appear later, in the zygomatic processes, nasal spine, and trochlear fossae. Some reports have stated that secondary ossification centers do not exist (Faro, Benoit, Wegrzyn, Chaoui, & Nicolides, 2005; Inman & Saunders, 1937). In any case, the calcification centers have completely fused by 6–7 months postconception. At birth, the frontal bones are separated by the frontal suture. Synostotic fusion of the frontal suture starts from around the second year of life and unites the frontal bones into a single bone by 7 years of age (Sperber, 2010).

The cartilaginous neurocranium initially comprises a number of separate cartilages (Sadler, 2012). Anterior to the hypophyseal fossa in the sella turcica, the prechordal cartilages are derived from neural crest cells to form the chondrocranium. The posterior part forms the chordal chondrocranium, arising from the paraxial mesoderm.

The ethmoid bone develops from two cartilages: the nasal capsule (ectehmoid) forms the cribriform plate and labyrinth, and the mesethmoid cartilage forms the perpendicular plate and crista galli (Sperber, 2010). The anterior part of the sphenoid body develops from presphenoid cartilage, and the lesser wings of the sphenoid bone develop from the orbitosphenoid cartilages (Kodama, 1965; Sperber, 2010).

### 4.2 Growth of the anterior cranial fossa in the fetus and juveniles

Growth of the anterior cranial fossa is accomplished by growth of the cartilage primordium itself and by growth between and within the synchondroses and the suture (Friede, 1981).

Growth of the anterior cranial fossa is more extensive in fetal life than after birth, and length increases more than width or height (Morimoto, Ogiwara, Kaytayama, & Shiota, 2008). The anterior cranial fossa forms later and grows more rapidly than the posterior cranial fossa before birth (Friede, 1981). At birth, most of the skull base is cartilaginous (Belden, Mancuso, & Kotzur, 1997; Hughes, Kaduthodil, Connolly, & Griffiths, 2010). Although growth of the anterior fossa is completed by 7 years of age, the posterior fossa continues to grow until puberty (Friede, 1981). Thus, the anterior cranial fossa forms earlier than the posterior.

The width of the anterior fossa increases by growth in (a) synchondroses of the presphenoid, (b) the frontal sutures, and (c) nasal capsule cartilage (Friede, 1981). These growth patterns are completed in the early stages, specifically (a) before birth, (b) by 2 years old, and (c) by 4 years old, respectively.

Increases in the length of the anterior fossa are accomplished by growth in (a) the nasal capsule cartilage, (b) the synchondroses of the presphenoid, (c) the fronto‐ethmoidal synchondroses (suture), (d) the spheno‐ethmoidal synchondroses (suture), and (e) the spheno‐frontal suture (Friede, 1981). Each of these is completed by 7 years of age, but bone resorption occurs in the orbital part of the frontal bone with the development of the frontal lobe of the brain.

The frontal fossa is completed by 7 years at the latest, but remodeling continues in the form of additions to the outer surface of the calvaria and resorption of the floor of the anterior fossa with the growth of the frontal brain (Friede, 1981; Melsen, 1974).

From the results of the present study, certain relationships seem to exist between the structure of the anterior cranial fossa in the adult and its development. The ethmoid spine develops markedly in some cases but poorly in others, that is, to almost the same level as the lesser wing of sphenoid. This result supports the findings of a previous study (van der Linden & Enlow, 1971). The ethmoid spine was clearly seen from the early development of the cranial base, and individual variations in anteroposterior diameter of the ethmoid spine appear to depend on individual differences from its first appearance.

The anterior part of the sphenoid bone is derived from the presphenoid (Sperber, 2010). This primordium has nine calcification centers, and the part forming the ethmoid spine shows the most delayed calcification (Kodama, 1965). Delayed calcification is considered one of the factors contributing to individual variations in ethmoid spine shape.

The intervention of the frontal bone appears with relatively low frequency, again supporting a previous study (van der Linden & Enlow, 1971). This structure is thought to appear when space is seen between the nasal capsule and the presphenoid at the time of occurrence. The difference in spatial space between the primordia forming each bone is thought to be involved to some extent in the final variations in bone morphology.

### 4.3 Morphological transitions with aging

**Figure 7:**
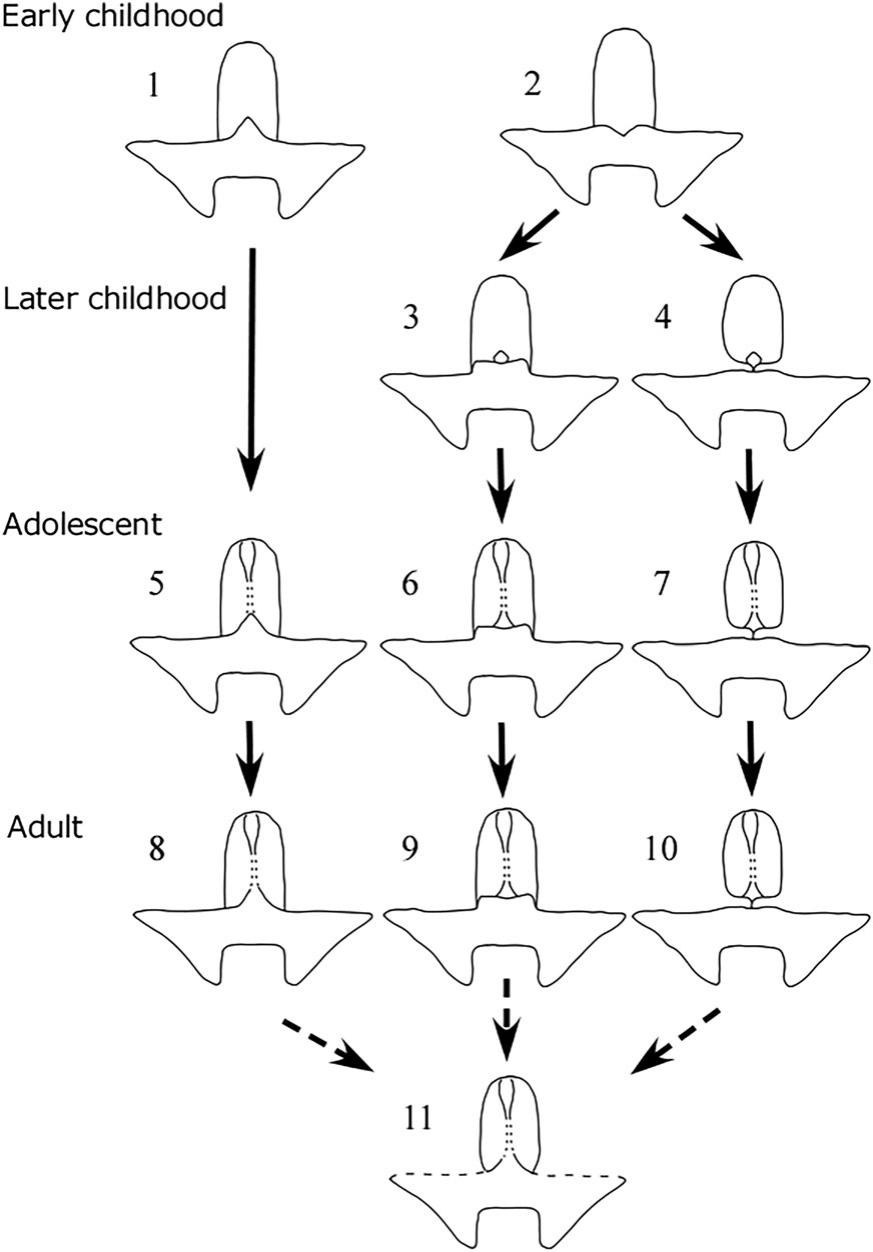
Postnatal changes in relationships among the three bones of the anterior cranial fossa. Solid arrows show growth change; dotted arrows show age change

Postnatal changes in relationships among the three bones of the anterior cranial fossa were discussed (Figure 7). In early childhood, an individual with a triangular ethmoid spine (Form 1) and another with a flat or slightly concave border between the ethmoid and sphenoid bones (Form 2) were seen. These two types changed with age.

Postnatal development of the form with the triangular ethmoid spine (Form 1) is expected to proceed as follows. Initially, a suture exists between the ethmoid and sphenoid bones, and the boundary is clear. As formation of the crista galli of the ethmoid bone progresses (Form 5), the ethmo‐sphenoidal suture disappears (Form 8). The suture of the skull are known to disappear by aging (Perizonius, 1984). The ethmo‐sphenoidal and fronto‐sphenoidal sutures also disappear with age (Dorandeu, 2008; Lingawi, 2012).

In infancy, the sphenoid bone is in contact with the ethmoid bone at a flat boundary (Form 2). The vertical height of the ethmoid bone is initially low but gradually increases. A small ossicle‐like structure appears at the boundary between the ethmoid and sphenoid bones (Forms 3 and 4). The ossicle is continuous with the crista galli, that is, part of the ethmoid bone. Orbital plates of the right and left sides of frontal bone extend towards the medial direction on the outside between the ethmoid and sphenoid bones. In the case of the formation of the frontal bone is active, the frontal bone penetrates between the ethmoid and the sphenoid bones (Form 4). Two situations are seen with further growth: one in which the ethmoid and sphenoid bones are in contact with each other without space (Form 6) and the second in which some space exists (Form 7). These two types are thought to grow while maintaining the respective forms (Forms 9 and 10). When the ethmo‐sphenoidal and fronto‐sphenoidal sutures and sutures of the right and left frontal bones disappear due to aging, the adult type with a triangular ethmoid spine is the result (Form 11). The juvenile skulls that we could observe were a few cases, and the above‐mentioned age change is a speculative process. Although the process may be modified by further research, we believe that it is reasonable hypothesis at this stage.

As mentioned above, morphological variations of the anterior cranial fossa in adults not only reflect fetal skull development and skull growth in childhood but also the effects of aging. Each bone of the anterior cranial base shows different developmental processes, and the initially minor differences are reflected in the morphological variations seen in adulthood.

## CONFLICT OF INTEREST

None declared.
